# Development of an Integrated Evaluation System for a Stretchable Strain Sensor

**DOI:** 10.3390/s16071114

**Published:** 2016-07-19

**Authors:** Hyungkook Jeon, Seong Kyung Hong, Seong J. Cho, Geunbae Lim

**Affiliations:** 1 Department of Mechanical Engineering, Pohang University of Science and Technology (POSTECH), 77 Cheongam-Ro, Nam-Gu, Pohang 790-784, Korea; likeblue@postech.ac.kr (H.J.); skhong@postech.ac.kr (S.K.H.); 2 School of Mechanical Engineering, Chungnam National University (CNU), 99 Daehak-Ro, Yuseong-Gu, Daejeon 305-764, Korea

**Keywords:** strain sensor, sensor evaluation system, flexible electronics, stretchable electronics

## Abstract

Recently, much research has been focused on stretchable or flexible electronic sensors for the measurement of strain or deformation on movable and variably shaped objects. In this research, to evaluate the performance of stretchable strain sensors, we have designed an integrated evaluation system capable of simultaneously measuring the change in stress and conductance of a strain sensor. Using the designed system, we have successfully evaluated the deformation characteristics, sensing range and sensing sensitivity of a stretchable strain sensor. We believe that the developed integrated evaluation system could be a useful tool for performance evaluation of stretchable strain sensors.

## 1. Introduction

Due to improvements in display technology, such as flexible screens and network communication technology, the portability and accessibility of electronic devices have increased, which has led to the continual development of wearable or attachable electronic devices [1,2,3,4,5,6,7,8,9,10,11,12,13]. Concerning the detection of human motion, many studies have addressed the development of stretchable strain sensors that can be attached directly onto the skin.

Highly stretchable and reversible systems, based on nanoscale structures, have attracted great interest in the field of stretchable strain sensors [3,6,7,8,9,11]. For example, Yamada et al. have introduced a highly stretchable strain sensor for measuring human motion using single-walled carbon nanotube films on a flat elastomeric poly(dimethylsiloxane (PDMS) substrate; the sensor has a capability of measuring strains up to 280% [7]. Nanosized structures, particularly carbon nanotubes, have been utilized to fabricate stretchable sensors with high sensitivity due to the critical surface effect at the nanoscale [7,14,15,16,17,18].

Most of the research on stretchable strain sensors has focused on measuring the change of conductance when the membrane is under an applied strain. As the stretchable strain sensors are applied with mechanical strain, their electrical property changes accordingly, which can be correlated back to the applied strain. These studies looked deep into the electrical properties of their suggested strain sensors. Surprisingly, mechanical property analysis of the strain sensors was missing, even in the works published in high impact journals [1,2,3,4,5,6,7,8,9]. Most of these stretchable strain sensors are composed of viscoelastic polymer membrane as the stretchable substrate. Viscoelastic polymers go through mechanical hysteresis during stretching process. The mechanical hysteresis of the sensors will obviously affect their electrical properties, which will lead to degradation of the sensing performances such as repeatability and durability. Because mechanical properties directly affect the electrical properties of the sensors, to properly evaluate the sensing performance of the strain sensors, it is clear that mechanical property analysis is necessary along with the ongoing electrical property analysis. 

Each of these studies has suggested a strain sensor with its own unique configurations [1,2,3,4,5,6,7,8,9]. Although their material and fabrication methods vary from one another, they share a similar application: motion detection of the human body. It is known that strains that occur in the human body are around 50%. For this reason, the strain sensors suggested in these studies are targeted to measure strains around 50%. To intuitively measure strains created on human skin, the sensors usually take form of thin films with few centimeters in length and the force applied to these sensors along with strain is around one newton (1 N).

To the best of our knowledge, there is no commercially sold set-up that is targeted at evaluating these strain sensors, which requires precisely measuring strains around 50%, forces around 1 N, while measuring the electrical resistance at the same time. Even if the electrical resistance measuring was disregarded, there is not an adequate performance evaluation set-up that is specifically designed to measure strains around 50% and forces around 1 N. Nano universal testing machines (UTMs) is currently available in the market, however the machine is built for small load tests and overly sophisticated with a resulting high price. The normal UTM for metal or polymer slabs is not suitable for thin membrane testing and the load range is set high for testing more rigid bodies. 

Therefore, in this research, we have designed an integrated evaluation system to measure changes in stress and conductance simultaneously under applied strain conditions. The evaluation system was controlled through a LabVIEW program that acquires information on applied strain, stress, and electrical resistance of the sensor simultaneously at desired rate. To evaluate the system, we fabricated a simple stretchable sensor comprising a thin metal layer and an elastomer substrate. By measuring stress and change of resistance across the strain sensor, we have successfully confirmed the sensor’s performance. We expect that the integrated evaluation system could be a useful tool for determining the material and electrical characteristics of conductive and stretchable strain sensors.

## 2. Materials and Methods

**Figure 1:**
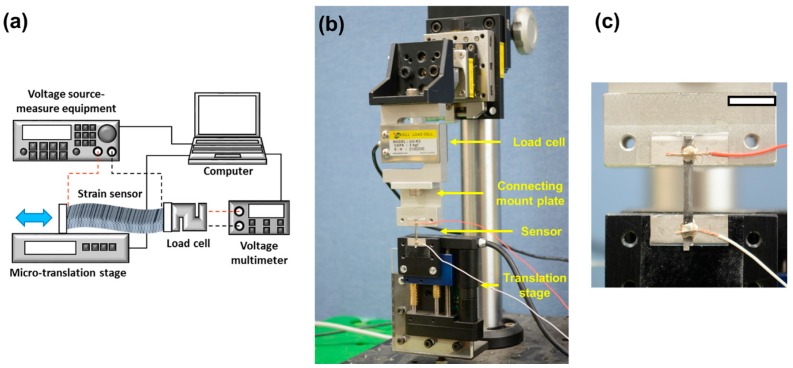
(**a**) Schematic diagram of the experimental setup, (**b**) photograph of experimental setup for measuring variation of stress and resistance under applied strain, (**c**) close up photograph of the stretchable strain sensor attached on the experimental system.

Figure 1 shows the experimental setup for measuring change of stress and conductance under an applied strain. The ends of the membrane were attached to the micro-translation stage (M-112, Physik Instrumente, Karlsruhe, Germany) and to the load cell (Model UU, Dacell, Cheongju, Korea), respectively. Most of the stretchable sensors possess nanostructures within or on the surface [1,2,3,4,5,6,7,8,9], which has to be protected for stable performance. Holding the sensor with mounting force will damage the nanostructure. Therefore a tightly-adhering double sided tape (DP 380, Shurtape, Avon, OH, USA) was chosen to affix the bottom surface of the fabricated membrane on the load cell and translation stage. Figure 1c shows how the fabricated membrane is attached on the experimental setup. In case extra force other than the adhesion force from the double sided tape was needed to keep the membrane attached, it was also designed for the membrane to be mounted on the translation stage and the load cell through screw in mounts. 

The strain applied to the membrane was controlled by the micro-translation stage with high resolution; the minimum incremental motion was 50 nm. The load cell consisted of Wheatstone bridge, which gives an output voltage dependent on the force applied to the load cell under a consistent input voltage. The specifications of the load cell features maximum load of 20 N, rated output of 1.5 mV/V, nonlinearity, hysteresis, and repeatability of 0.03% of the rated output. The load cell was suggested to perform best under 10 V of actuation voltage. Under 10 V, 0.03% of the rated output equals to 4.5 μV in terms of voltage and 6 mN in terms of the applied load. The output voltage from the load cell was measured using a high-resolution multimeter (34401A, Agilent, Santa Clara, CA, USA), with an accuracy within 10^−6.5^ V of reading which is enough to read 4.5 μV, the voltage resolution of the load cell. 

The change of conductance and resistance of the membrane was determined using the current across the membrane, measured by a voltage-source-measure equipment (B2902A, Keysight, Santa Rosa, CA, USA), which had a minimum resolution of 100 fA, under a constant voltage of 1 V.

We created a LabVIEW program to control the translation stage and simultaneously obtain the data from the voltage-source-measure unit and the multimeter. In the LabVIEW program, the desired voltage was applied to the membrane through the voltage-source-measure equipment and controlled the movement of the translation stage with desired displacement, speed, and cycling number. While applying a constant voltage but increasing the length of the membrane, the change in current across the membrane was measured by the voltage-source-measure equipment, and the potential change of the load cell was measured by the multimeter, simultaneously. Finally, the applied strain and the change of stress and resistance of the membrane was measured in real time.

**Figure 2:**
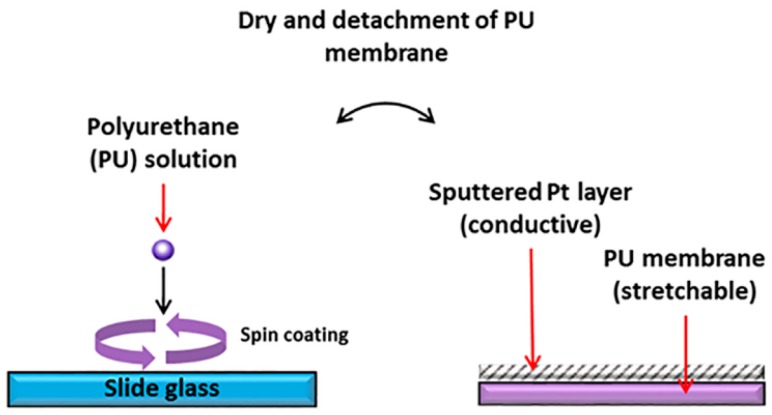
Fabrication process of the stretchable and conductive membrane.

To validate the integrated evaluation system, we fabricated a simple strain sensor consisting of a conductive metal layer and stretchable membrane, as shown in Figure 2. First, a thermoplastic polyurethane (PU) (Pellethane 2363-80AE, Lubrizol, Wickliffe, OH, USA) was dissolved in a 13 wt% mixture of tetrahydrofuran (THF) and dimethylformamide (DMF, 60/40, v/v). 

Then, a spin-coating of the PU solution was applied to a glass slide. The spinning speed controlled the thickness of the PU membrane (thickness of 23 μm at 500 rpm). Following that, a thin layer of platinum (Pt) was applied onto the PU membrane by a conventional sputtering method. Finally, the PU membrane was cut into 3 cm long strips with a width of 2 mm. The fabricated membrane exhibited both stretch-ability and conductivity. After the fabrication of membrane was completed, conductive epoxy (CW 2400, Chemtronics, Kennesaw, GA, USA) was applied on each end of the Pt surface to fasten electrical wirings which was later plugged into the voltage-source-measure equipment to measure the resistance of the Pt layer.

## 3. Results and Discussion

### 3.1. Specifications of the Developed Evaluation System

The developed evaluation system is targeted to evaluating the performance of stretchable strain sensors. The sensors share a common application field, human motion detection, and it is known that strains that occur in the human body are around 50%. The evaluation system requirements are as follows: precisely measuring of strain around 50%, load around 1 N, firmly attaching of thin strip membrane, and measuring of the electrical resistance. The developed system features appropriate load range with reasonable load resolution. The extension resolution of 50 nm. The system is adequate for precisely measuring strains around 50% applied on few centimeter long sensors, which is the desired target for stretchable sensor evaluation. 

Table 1 summarizes and compares the general specifications of the Nano UTM and UTM with the developed system. In case of Nano UTM, the maximum load is too low for evaluating stretchable strain sensors. Beside from the maximum load, other specifications are too sophisticated which results in high price of the system. In case of the UTM, the load range is designed for high load metal or polymer slab testing which is inadequate for thin membrane sensor testing. Also the extension resolution is expected to be blunt for incremental measuring of the strain applied to the sensors. Nano UTM and UTM might have superior specifications in specific areas. However, our developed system provide a more adequate evaluation performance overall when evaluating stretchable strain sensors.

### 3.2. Deformation Characteristics of the Developed Membrane

**Figure 3:**
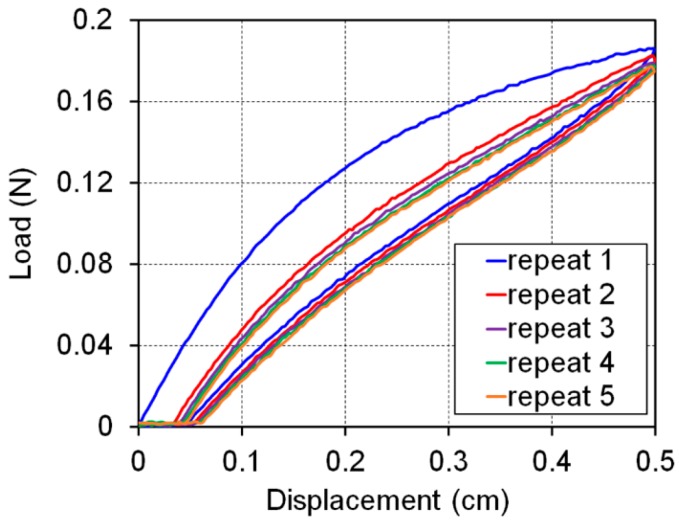
Change in the stress applied to the fabricated PU membrane depending on the strain (stage moving speed: 100 μm/s).

A repetition test was undertaken to evaluate the deformation characteristics of the fabricated PU membrane, stretching and shrinking the membrane with a maximum strain of 50% at a stage speed of 100 μm/s (Figure 3). 

From the data presented in Figure 3, the strain was calculated by dividing the displacement (from initial position of the stage) by the initial length of the membrane (1 cm). The tensile engineering stress was calculated by dividing the measured load from the load cell by the cross-sectional area of the membrane (0.046 mm^2^).

The membrane did not recover to the initial length, as shown in Figure 3, indicating plastic deformation (about 10%). During the second test, the stress was considered to be zero until the applied strain was over 10%. This means that no force was experienced by the membrane at strains under 10% because of the plastic deformation. In fact, as the number of repetitions increased, the stress curve indicated saturated stress behavior because the plastic deformation also reached a saturated state. The plastic deformation behavior and the saturation stress curve are in accordance with the general characteristics of elastomers such as PU. The integrated experimental setup was able to measure the load applied to the strain sensor with a very high resolution (~6 mN). This highly sensitive evaluation system can be used to measure very small forces applied to materials with low mechanical strength. The evaluation of the hysterical behavior will be revisited in the next section.

### 3.3. Analysis of Resistance Change of the Developed Membrane Depending on Applied Strain

**Figure 4:**
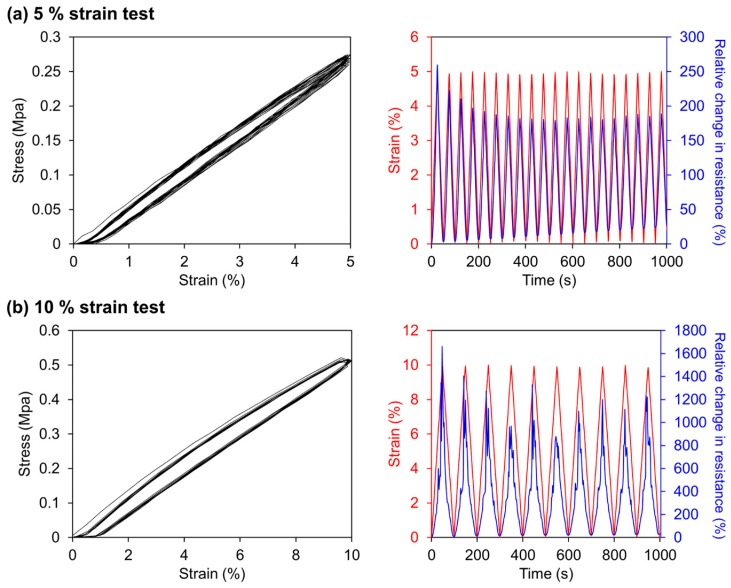
Stress-strain curve and the relative change in resistance during cycling between (**a**) 0%~5% strain and (**b**) 0%~10% strain (stage moving speed: 100 μm/s).

Figure 4 shows the stress-strain curve and the relative change in resistance (=Δ*R*/*R_off_*, where *R* and Δ*R* = *R_on_* – *R_off_* denote the resistance and the resistance difference, respectively) during strain cycling between 0%~5% and 0%~10%. In this case, *R_on_* indicates the resistance of the stretched membrane at any given strain and *R_off_* indicates the initial resistance of the membrane at 0% strain. The results show that the membrane had a very high gauge factor (*G =* (Δ*R*/*R_off_*)/*ε* ~ 50, where *ε* denotes the applied strain compared to conventional stretchable strain sensors [1,2,3,4,5,6,7,8,9,10], which denotes the high sensitivity of our membrane.

In the stress-strain curves for both strain tests in Figure 4, there exists an apparent strain hysteresis which is caused by the plastic deformation of the membrane. As the strain cycle continued, the hysteresis was saturated up until one tenth of the maximum strain applied during the cycles, namely 0.5% for 5% strain test and 1% for 10% strain test. The right graph of Figure 4a shows repetitive tests of both strains and the accordingly acquired relative change in resistance. As the sensor went through the strain cycles, the lower peaks of relative change in resistance gradually grew from zero. This is the result of the plastic deformation that prevents the sensor to return to its original electrical resistance. 

It is clearly demonstrated that the strain hysteresis caused by plastic deformation of the sensor affects the electrical resistance and ultimately the performance of the sensor. The results suggest that mechanical properties of the stretchable strain sensors have to be analyzed to properly evaluate their performance. With the evaluation system suggested in this work, these stretchable strain sensors can be properly analyzed thus providing a deeper understanding of the sensor performance.

**Figure 5:**
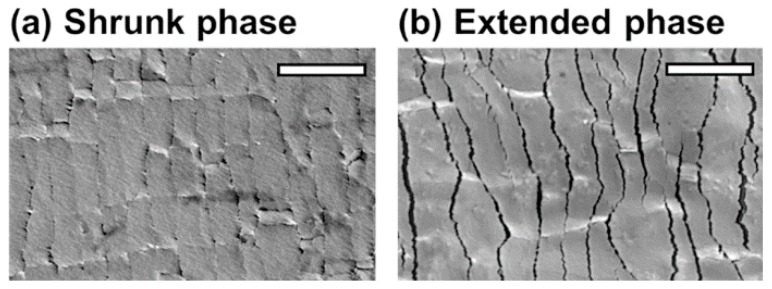
High-resolution scanning electron microscopy image of the cracks in the platinum (Pt) layer during (**a**) shrunk phase and (**b**) extended phase (5% strain) (Scale bar: 3 μm).

The change of resistance was caused by the change of electrical connection due to formation of cracks on Pt layer as strain is applied to the sensor. As the membrane was stretched, cracks were generated in the Pt layer. The spacing and shape of the cracks varied depending on the strain applied to the membrane, which affected the membrane’s resistance. Figure 5a shows the shrunk phase of the sensor where the cracks are closed while Figure 5b shows the extended phase where the cracks are opened up due to the applied strain in the direction of white arrows. 

When a larger strain was applied to the membrane strain (10%), the resistance was observed to increase dramatically as shown in Figure 4b. The peaks of relative change in resistance is not consistent throughout the strain cycles and show much higher values compared to the 5% strain cycles. This might have been caused by the Pt layer dramatically losing its conductance due to the wide cracks formed on its surface. It can be inferred from the results that the maximum strain the fabricated sensor can measure is under 10%.

## 4. Conclusions

In this research, an integrated evaluation system for the performance evaluation of a stretchable strain sensor has been developed. Using the system, we have simultaneously measured the change of stress and resistance under an applied strain to a conductive and stretchable membrane. To validate the evaluation system, a simply structured, conductive and stretchable membrane was fabricated to act as a strain sensor. Results confirmed the deformation characteristics and the electrical characteristics of the strain sensor under a cyclic stretching test. Furthermore, the mechanism of reversible resistance change of the membrane was identified as the formation of cracks in the Pt layer. We expect that the developed integrated evaluation system would be a useful tool for evaluating the sensing performance of stretchable strain sensors, promoting the development of novel stretchable strain sensors with high performance.

## Figures and Tables

**Table 1:** General specifications of Nano UTM, UTM, and the developed system.

	Nano UTM	UTM	Developed System
Maximum Load	0.5 N	50~5000 N	20 N
Load Resolution	50 nN	200 μN	6 mN
Extension Resolution	35 nm	Not Available	50 nm
Extension Rate	~5 mm/s	0.01~500 mm/min	~1.5 mm/s
Resistance Measurement	X	X	O

Comparing the general specifications of Nano UTM, UTM, and the developed system.
